# Brain Network Modularity Predicts Exercise-Related Executive Function Gains in Older Adults

**DOI:** 10.3389/fnagi.2017.00426

**Published:** 2018-01-04

**Authors:** Pauline L. Baniqued, Courtney L. Gallen, Michelle W. Voss, Agnieszka Z. Burzynska, Chelsea N. Wong, Gillian E. Cooke, Kristin Duffy, Jason Fanning, Diane K. Ehlers, Elizabeth A. Salerno, Susan Aguiñaga, Edward McAuley, Arthur F. Kramer, Mark D'Esposito

**Affiliations:** ^1^Helen Wills Neuroscience Institute, University of California, Berkeley, Berkeley, CA, United States; ^2^Beckman Institute for Advanced Science and Technology, University of Illinois at Urbana-Champaign, Urbana, IL, United States; ^3^Department of Psychological and Brain Sciences, University of Iowa, Iowa City, IA, United States; ^4^Department of Human Development and Family Studies, Colorado State University, Fort Collins, CO, United States; ^5^Interdisciplinary Health Sciences Institute, University of Illinois at Urbana-Champaign, Urbana, IL, United States; ^6^Department of Kinesiology and Community Health, University of Illinois at Urbana-Champaign, Urbana, IL, United States; ^7^Department of Internal Medicine-Gerontology, Wake Forest School of Medicine, Winston-Salem, NC, United States; ^8^Psychology Department and Mechanical and Industrial Engineering Department, Northeastern University, Boston, MA, United States

**Keywords:** executive function, cognitive control, functional connectivity, exercise, brain network modularity

## Abstract

Recent work suggests that the brain can be conceptualized as a network comprised of groups of sub-networks or modules. The extent of segregation between modules can be quantified with a modularity metric, where networks with high modularity have dense connections within modules and sparser connections between modules. Previous work has shown that higher modularity predicts greater improvements after cognitive training in patients with traumatic brain injury and in healthy older and young adults. It is not known, however, whether modularity can also predict cognitive gains after a physical exercise intervention. Here, we quantified modularity in older adults (*N* = 128, mean age = 64.74) who underwent one of the following interventions for 6 months (NCT01472744 on ClinicalTrials.gov): (1) aerobic exercise in the form of brisk walking (Walk), (2) aerobic exercise in the form of brisk walking plus nutritional supplement (Walk+), (3) stretching, strengthening and stability (SSS), or (4) dance instruction. After the intervention, the Walk, Walk+ and SSS groups showed gains in cardiorespiratory fitness (CRF), with larger effects in both walking groups compared to the SSS and Dance groups. The Walk, Walk+ and SSS groups also improved in executive function (EF) as measured by reasoning, working memory, and task-switching tests. In the Walk, Walk+, and SSS groups that improved in EF, higher baseline modularity was positively related to EF gains, even after controlling for age, in-scanner motion and baseline EF. No relationship between modularity and EF gains was observed in the Dance group, which did not show training-related gains in CRF or EF control. These results are consistent with previous studies demonstrating that individuals with a more modular brain network organization are more responsive to cognitive training. These findings suggest that the predictive power of modularity may be generalizable across interventions aimed to enhance aspects of cognition and that, especially in low-performing individuals, global network properties can capture individual differences in neuroplasticity.

## Introduction

Aging is accompanied by changes in cognition and brain function, yet there is individual variability in the extent to which older adults experience such effects (Wilson et al., 2002; Raz et al., 2005; Fabiani, 2012; Burzynska et al., 2015; Salthouse, 2016). Individual differences in age-related cognitive decline, particularly in executive function processes, are related to changes in structural and functional connectivity between brain regions (Andrews-Hanna et al., 2007; Damoiseaux et al., 2008; Kennedy and Raz, 2009; Madden et al., 2009, 2012). One method to quantify these complex interactions is to conceptualize the brain as a network comprised of sub-networks, or modules (Newman and Girvan, 2004; Newman, 2006b; Chen et al., 2008; Bullmore and Sporns, 2009; Meunier et al., 2010; Betzel et al., 2014; Bertolero et al., 2015). The extent of a module's segregation from the rest of the network can be quantified with a modularity metric (Newman and Girvan, 2004), where networks with high modularity have many connections within modules and fewer connections between modules. Computational models suggest that a modular network organization allows for a system that is more adaptable to new environments (Kashtan and Alon, 2005; Clune et al., 2013; Tosh and McNally, 2015), suggesting a role for network modularity in supporting complex behaviors like executive function. Compared to young adults, older adults have less modular brain networks (Chen et al., 2011; Onoda and Yamaguchi, 2013; Betzel et al., 2014; Geerligs et al., 2015) with pronounced age-related differences in sub-networks that support “associative” processes, such as executive function (Chan et al., 2014). Taken together, these findings suggest that more modular brain networks enable complex cognitive processes and neuroplasticity and, further, may provide insight into the mechanisms underlying the effectiveness of interventions geared toward ameliorating age-related cognitive decline.

Recent work has demonstrated that individual differences in brain network modularity can predict the extent to which individuals improve after cognitive interventions aimed to improve executive function. Specifically, higher baseline modularity (i.e., measured prior to the intervention) quantified during a task-free “resting state” predicted greater improvements after cognitive training in patients with traumatic brain injury (Arnemann et al., 2015) and more recently, in healthy older (Gallen et al., 2016) and young adults (Baniqued et al., 2015). Importantly, modularity predicted training gains even after controlling for baseline cognitive performance. These findings suggest that the informative nature of such individual differences in brain network organization can be used to maximize intervention effectiveness, such as by modifying training intensity or duration, especially in populations where behavioral measures may be difficult to collect (Gabrieli et al., 2015). Previous studies have examined other neural metrics in relation to learning and training responses (Erickson et al., 2010; Basak et al., 2011; Vo et al., 2011; Mathewson et al., 2012), but have often focused on specific brain regions related to specific types of interventions. As modularity has been shown to be reliable in individuals across sessions (Stevens et al., 2012; Cao et al., 2014) and predictive of cognitive gains across a variety of populations and training protocols, modularity may be a unifying biomarker that indexes an individual's potential for adaptive reorganization with intervention.

In addition to cognitive training interventions, cost-effective and easily accessible physical activity interventions involving brisk walking have been shown to have rehabilitative and protective effects on brain function in older adults (Kramer et al., 2006; Voss et al., 2013c). Further, there are significant individual differences in responsiveness to exercise training, with factors such as initial levels of heart rate and blood pressure determining gains in cardiorespiratory fitness (Bouchard and Rankinen, 2001). Although we have previously found that individual differences in brain network modularity can predict training-related gains after cognitive training (Arnemann et al., 2015; Baniqued et al., 2015; Gallen et al., 2016), it is not yet known whether the relationship between modularity and training gains is generalizable to interventions aimed to enhance executive function in older adults. Although there are several graph theoretical metrics, we were specifically interested if this relationship between pre-intervention brain modularity and training gains can also be found in a different, non-cognitive training intervention, such as a physical exercise intervention.

Specifically, we hypothesize that modularity reflects an individual's readiness to engage in and benefit from training. A recent study demonstrated that individuals with higher general intelligence show smaller connectivity changes between a resting state and task states, suggesting the existence of a more “optimal” network organization that provides more efficient reconfiguration during performance of various tasks (Schultz and Cole, 2016). Similar to this idea, we hypothesize that a more optimal—more modular network configuration is better able to transition to task states demanded by the interventions; it is more adaptable. In the context of the current study, a more modular brain network may potentiate the rehabilitative and protective effects of physical exercise on the aging brain, leading to greater improvements in executive function.

Here, we examined brain network modularity in older adults who underwent a 6-month exercise training intervention. Specifically, we tested the hypothesis that higher baseline modularity predicts larger exercise-related gains in cognition. The current study employed a broad battery of cognitive tests to assess intervention-related gains in executive function, episodic memory, vocabulary and perceptual speed. Here, we focused on the relationship between baseline modularity and improvements in executive function, as these processes show pronounced age-related decline and exercise-related changes (Hillman et al., 2008; Voss et al., 2013c; Kawagoe et al., 2017).

## Materials and methods

### Participants

Healthy, low active, older adults (*N* = 247) aged 60–80 from the Urbana-Champaign community participated in a randomized controlled exercise trial (https://clinicaltrials.gov/ct2/show/NCT01472744; see Voss et al., 2016; Burzynska et al., 2017; Fanning et al., 2017; Ehlers et al., 2017a,b, for data published from this same cohort). All participants provided informed consent and the University of Illinois Institutional Review Board approved all procedures used in the study. Selection criteria consisted of the following, (1) >75% right-handed on the Edinburgh Handedness Questionnaire; (2) normal or corrected-to-normal vision of at least 20/40; (3) no color-blindness; (4) no history of stroke, transient ischemic attack, or head trauma; (5) >23 score on Mini-Mental State Examination (MMSE); (6) >21 score on Telephone Interview of Cognitive Status (TICS); (7) <10 score on Geriatric Depression Scale (GDS); (8) reported that they engaged in moderate intensity exercise for 30+ min no more than twice a week in the last 6 months and 9) screened for safe participation in an MRI environment (e.g., no claustrophobia or metallic implants). In all analyses presented here, we further excluded participants with MMSE scores less than 27 (*N* = 26), as a more stringent criterion is recommended in highly educated samples such as in the current study (O'Bryant et al., 2008). Summary demographics for included participants are provided in Table 1. Additional data were excluded on a case-by-case basis during data quality procedures applied to each behavioral measure. Specifically, cognitive measures greater than 3 SD from the mean were excluded. After this step, to reduce the influence of remaining extreme values, scores greater than 3 SD from the recomputed mean were winsorized (Tukey, 1962; Wilcox, 2005) to the appropriate cut-off value (3 SD below or above the mean). Analyses involving only fitness or behavioral scores were performed on the larger sample (*N* = 188), prior to exclusion due to MRI data quality, but effects were similar in the MRI sample (*N* = 128).

**Table 1:** Demographics.

	**Walk**	**Walk+**	**Dance**	**SSS**	**Group effect**
FULL SAMPLE^*^					
Age	64.82 (4.55), 60–77	64.51 (4.47), 60–78	65.58 (4.57), 60–78	65.52 (4.43), 60–78	*F*_(3, 217)_ = 0.75, *p* = 0.53
Education	16.13 (3.31), 12–26	15.60 (2.26), 12–20	15.54 (3.17), 12–25	16.44 (3.14), 12–26	*F*_(3, 217)_ = 1.19, *p* = 0.31
MMSE	28.86 (1.02), 27–30	28.80 (0.94), 27–30	28.90 (0.94), 27–30	28.94 (1.08), 27–30	*F*_(3, 217)_ = 0.20, *p* = 0.90
VO_2_peak	19.97 (5.11), 12–34	19.78 (4.15), 9–28	19.98 (4.69), 10–33	19.22 (4.73), 6–30	*F*_(3, 215)_ = 0.35, *p* = 0.79
Female	34	33	42	40	$\chi_{\left(3\right)}^{2}$ = 0.18, *p* = 0.98
N	49	49	59	64	221
**BEH + CRF**					
Age	64.64 (4.06), 60–75	64.80 (4.49), 60–77	65.50 (4.50), 60–78	65.81 (4.43), 60–78	*F*_(3, 184)_ = 0.78, *p* = 0.51
Education	16.43 (3.30), 12–26	15.74 (2.21), 12–20	15.52 (3.12), 12–25	16.70 (3.22), 12–26	*F*_(3, 184)_ = 1.70, *p* = 0.17
MMSE	28.93 (1.02), 27–30	28.82 (0.95), 27–30	28.92 (0.94), 27–30	28.89 (1.11), 27–30	*F*_(3, 184)_ = 0.11, *p* = 0.96
VO_2_peak	20.21 (5.04), 12–34	19.76 (4.06), 9–28	19.99 (4.07), 13–29	19.53 (4.69), 7–30	*F*_(3, 182)_ = 0.20, *p* = 0.90
Female	28	29	34	36	$\chi_{\left(3\right)}^{2}$ = 0.32, *p* = 0.96
N	42	44	48	54	188
**MRI SAMPLE**					
Age	63.83 (3.96), 60–75	64.59 (4.27), 60–77	65.06 (4.00), 60–73	65.29 (4.18), 60–75	*F*_(3, 124)_ = 0.78, *p* = 0.51
Education	16.59 (2.80), 12–24	15.66 (2.31), 12–20	15.78 (3.16), 12–26	17.22 (3.40), 12–26	*F*_(3, 124)_ = 2.06, *p* = 0.11
MMSE	29.07 (0.92), 27–30	28.86 (0.92), 27–30	28.94 (0.98), 27–30	29.08 (1.08), 27–30	*F*_(3, 124)_ = 0.36, *p* = 0.78
VO_2_peak	21.60 (5.39), 12–34	20.12 (4.14), 11–28	20.11 (4.00), 13–27	19.70 (4.60), 7–30	*F*_(3, 122)_ = 1.02, *p* = 0.39
Female	18	19	23	27	$\chi_{\left(3\right)}^{2}$ = 0.93, *p* = 0.82
N	29	29	32	38	128

*Mean (SD) and range for age, education, MMSE and VO_2_peak*.

* *Full sample excludes participants with MMSE scores lower than 27. Two participants are missing VO_2_peak data*.

For the MRI data, we excluded one participant with incomplete resting state data, one participant with structural abnormalities (see section MRI Acquisition and Processing for more details), and 39 participants who reported taking medications known to influence the central nervous system. Thirty-five participants whose resting state scans contained more than 10% of volumes with movement greater than 0.50 framewise displacement (FD) or any volume with a maximum absolute displacement of 4.0 mm were excluded. MRI data were not collected for five subjects. Demographics for this reduced sample are provided in Table 1.

### Protocol summary

All participants underwent MRI, behavioral, and fitness testing sessions before and after a 6-month long physical exercise intervention. Participants were paid for the pre- and post-testing sessions at a rate of $10/h. Participants were randomly assigned to one of four intervention groups, which met for an hour three times a week. All group sessions were led by trained exercise specialists. In the walking group (Walk), participants were instructed to walk within their target heart rate (50–60% of their maximal heart rate for first 6 weeks, 60–75% for last 18 weeks). A second group was also instructed to walk within the same target heart rate and was provided with a daily milk-based supplement formula provided by Abbott Nutrition that contained beta-alanine (Walk+). A third group was instructed in exercises focusing on stretching, strengthening and stability (SSS). A fourth group (Dance) was instructed in social dance sequences (i.e., Contra and English country dancing) by experienced dance instructors. Since the focus of this study is on the utility of brain modularity in predicting intervention-related gains, we limit our discussion of the intervention approach and choice of training regimen (for detailed information, see Ehlers et al., 2016; Burzynska et al., 2017).

### Cardiorespiratory fitness testing

Participants underwent cardiorespiratory fitness (CRF) testing before and after the intervention. CRF reflects the integrated ability of the cardiovascular and respiratory systems to deliver oxygen during sustained physical effort (Ross et al., 2016), and regular physical exercise increases the efficiency of these systems (Wenger and Bell, 1986). CRF testing involves gradually increasing exercise intensity to tax the aerobic system and measuring the corresponding increase in oxygen consumption. Physician's approval was solicited prior to testing. CRF, operationally defined as peak oxygen consumption (VO_2_peak in mL/kg/min, relative rate in milliliters of oxygen per kilogram of body mass per minute), was measured with indirect calorimetry during a modified Balke graded maximal exercise test on a motor-driven treadmill (Balke and Ware, 1959; Froelicher et al., 1975). Participants walked on a treadmill at a constant pace while the incline was increased 2–3% every 2 min. Expired air was sampled at 30-s intervals until maximal VO_2_ was reached or the test was terminated due to volitional exhaustion and/or symptom limitation. Maximal VO_2_ was determined after two of three criteria were met: (1) a plateau in VO_2_ after increase in workload; (2) a respiratory exchange ratio (ratio of CO_2_ production and O_2_ consumption, reflecting limits of cardiovascular system) >1.10, and (3) a maximal heart rate within 10 bpm of their age-predicted maximum. VO_2_peak was the highest VO_2_ recorded during the test. For the correlation analyses, we calculated a standardized CRF gain score for each individual by taking the difference between post-and pre-scores and dividing this by the standard deviation of pre-test scores (SD collapsed across groups).

### Behavioral testing

Participants underwent cognitive testing before and after the interventions. With the exception of the Switching Task and the Spatial Working Memory Task, all tests were taken from the Virginia Cognitive Aging Project (VCAP) (Salthouse and Ferrer-Caja, 2003; Salthouse, 2004, 2005, 2010). The VCAP tests were categorized into four categories: vocabulary, perceptual speed, episodic memory, and fluid reasoning. In the analyses, we grouped the Switching Task and Spatial Working Memory Task together with the fluid reasoning tasks to create an “executive function” component score, given previously demonstrated relationships between cognitive control and fluid reasoning abilities (Kane et al., 2005; Salthouse, 2005). We also performed a principal components analysis (PCA) on all the pre-test measures to confirm the VCAP construct groupings and to confirm that the Switching and Spatial Working Memory Tasks were related to performance on the fluid reasoning tests (Table 2, Supplementary Table 1). For each pre-test and post-test measure, we calculated standardized scores (z-scores) and averaged these z-scores according to the task groupings specified above, resulting in four component scores representing baseline cognitive abilities in vocabulary, perceptual speed, episodic memory and executive function (fluid reasoning plus switching and working memory). For each test, we also calculated standardized gain scores by subtracting pre-test performance from post-test performance, and dividing this value by the standard deviation of raw pre-test scores (collapsed across groups). We averaged the standardized gain scores accordingly to create composite gain scores in vocabulary, perceptual speed, episodic memory, and executive function. The following sections have brief descriptions of each test and the specific measure used for analyses.

**Table 2:** PCA standardized loadings (pattern matrix) based upon correlation matrix of baseline scores.

	**PC1**	**PC2**	**PC4**	**PC3**
Digit symbol	0.19	*0.85*	0.07	0.17
Pattern comparison	0.19	*0.79*	0.07	0.05
Letter comparison	0.01	*0.83*	0.18	0.02
Word recall	−0.01	0.23	0.34	*0.75*
Logical memory	0.23	0.16	0.34	*0.69*
Paired associates	0.18	0.04	0.12	*0.83*
Shipley abstraction	*0.56*	0.30	0.46	0.27
Form boards	*0.69*	0.28	0.21	0.00
Letter sets	*0.50*	0.40	0.38	0.25
Matrix reasoning	*0.65*	0.26	0.28	0.29
Paper folding	*0.77*	0.05	0.13	0.18
Spatial relations	*0.85*	0.20	0.15	0.04
Word vocabulary	0.13	0.13	*0.85*	0.25
Picture vocabulary	0.39	0.02	*0.74*	0.11
Synonym-antonym	0.22	0.04	*0.82*	0.24
Spatial working memory	*0.29*	0.53	0.03	0.12
Task switching bin score	*−0.23*	−0.44	0.12	−0.33

*Performed varimax rotation and extraction of 4 components, which accounted for 67% of total variance. Italics denote component groupings*.

#### Task-switching (Kramer et al., 1999; Voss et al., 2010a,b, 2013b; Leckie et al., 2014)

On each trial, participants were shown a number between 1 and 9 (except 5) against a colored background: (1) on a pink background, participants were instructed to determine whether the number was odd or even, (2) on a blue background, they were to determine if the number was higher or lower than 5. Participants completed a high/low practice block (40 trials) an odd/even practice block (40 trials), a single high/low task block (40 trials), a single odd/even task block (40 trials), a mixed practice block (64 trials) and a mixed task block (160 trials). We analyzed performance on the mixed task block and extracted (1) local switch cost (mixed switch reaction time; RT—mixed non-switch RT) and (2) task switching bin score (combination of accuracy and RT measures) (Draheim et al., 2016). The task switching bin score was used in the principal components and correlation analyses to better examine the relationship between task switching performance and performance on other tests (Draheim et al., 2016). Local RT switch cost was used in the analyses of intervention effects, consistent with previous studies (Voss et al., 2010a, 2013b). The two measures were correlated (Supplementary Table 1; baseline measures: *r*_(211)_ = 0.322, *p* < 0.001, two-tailed; standardized gain scores: *r*_(159)_ = 0.267, *p* < 0.001, two-tailed), and the intervention effects were similar when using bin score instead of local RT switch cost.

#### Spatial working memory (Erickson et al., 2011)

On each trial, an arrangement of two, three, or four black dots was briefly presented on the screen. After a delay, a red dot appeared and participants were instructed to determine if the red dot matched the position of one of the black dots presented earlier in that trial (match or non-match). Participants performed a practice block of 12 trials, and a task block of 120 trials (40 trials per condition). We analyzed mean accuracy during the task block for the more difficult three-dot and four-dot trial conditions.

#### Shipley abstraction (Zachary, 1986)

Participants were given a list of word, letter, or number sequences on a piece of paper and were instructed to write the missing item/s (word, letter or number) in each sequence. Participants were given 5 min to answer 20 items. We analyzed the total number of correctly answered items.

#### Matrix reasoning (Ravens, 1962)

On each trial, participants were shown a 3 × 3 grid, with each cell except for one containing an abstract pattern. Participants were instructed to select which among eight options best completes the matrix along both the rows and columns. Participants performed two practice trials and were then given 10 min to complete a maximum of 18 items. We analyzed the total number of correctly answered items.

#### Paper folding (Ekstrom et al., 1976)

On each trial, participants were presented with images that show a sheet of paper folded in a certain sequence and a hole punched through the folded sheet. Participants were asked to select which among five options matched the pattern of holes that would result when the paper was unfolded. They were given 10 min to complete a maximum of 12 trials. We analyzed the total number of correctly answered items.

#### Spatial relations (Bennett et al., 1997)

On each trial, participants were presented with a 2-dimensional object pattern and instructed to identify which among four three-dimensional figures would match the 2-dimensional pattern when folded. Participants were given 10 min to complete a maximum of 20 trials. We analyzed the total number of correctly answered items.

#### Form boards (Ekstrom et al., 1976)

On each trial, participants were presented with a specific shape and instructed to choose which pieces (five total options) will exactly fill the space inside the shape. They were given 8 min to complete a maximum of 24 trials. We analyzed the total number of correctly answered items.

#### Letter sets (Ekstrom et al., 1976)

On each trial, participants were presented with five sets of four-letter strings and asked to determine which set was different from the other four. Participants were given 10 min to complete a maximum of 15 trials. We analyzed the total number of correctly answered items.

#### Digit-symbol coding (Wechsler, 1997a)

Participants were presented with a sheet of paper containing a series of numbers between 1 and 9, were asked to fill in the corresponding symbol based on a digit-symbol key provided. Participants completed 7 practice items and were given 2 min to complete a maximum of 133 items. We analyzed the number of correctly answered items.

#### Pattern comparison (Salthouse and Babcock, 1991)

Participants were given a sheet of paper with a set of line patterns and were tasked to determine whether a pair of line patterns was the same or different. Participants completed three practice items, followed by two task sets, each set with a maximum number of 30 items to be completed within 30 s. We analyzed the number of correctly answered items, averaged across two sets of problems.

#### Letter comparison (Salthouse and Babcock, 1991)

Participants were given a sheet of paper with a set of non-word letter strings and were tasked to determine whether a pair of letter strings was the same or different. Participants completed three practice items, followed by two task sets, each set with a maximum number of 30 items to be completed within 30 s. We analyzed the number of correctly answered items, averaged across two sets of problems.

#### Logical memory (Wechsler, 1997b)

Participants listened to stories narrated by an experimenter and after each reading, were asked to recall each story in detail. We analyzed the number of correctly recalled story details, summed across three story-tellings (first story, second story, re-reading of second story).

#### Paired associates (Salthouse et al., 1996)

Participants listened to a list of six word pairs read aloud by an experimenter. The experimenter then read the first word of each pair and asked participants to recall the paired second word. We analyzed the number of correctly recalled items, averaged across two sets of six pairs each.

#### Word recall (Wechsler, 1997b)

Participants listened to a list of words and were given 90 s to recall the words in any order. Participants listed to the same list three more times and were asked to recall as many words as possible after each reading. Participants were then read a new list of words, asked to recall as many words as possible from the new list, and then asked to recall words from the old list. We analyzed the total number of correctly recalled items.

#### Word vocabulary (Wechsler, 1997a)

Experimenters read aloud a list of 33 words and asked participants to verbally give the meaning of each word. Responses are scored 0–2 points according to the quality of the definition (based on provided word and phrase guidelines). The test is discontinued after six consecutive scores of 0. We analyzed the total number of points.

#### Picture vocabulary (Woodcock and Johnson, 1989)

Experimenters present a maximum of 30 images and participants are tasked to name the objects presented. The test is discontinued after a participant fails to name six consecutive items. We analyzed the total number of correctly named items.

#### Synonym-antonym (Salthouse, 1993)

On each trial, participants are presented a target word and are tasked to select which among five word options is most similar (synonym) or opposite (antonym) in meaning to the target word. Participants completed a synonym block followed by an antonym block, each with a maximum of 10 items to be completed within 5 min. We analyzed the total number of correctly identified words across the synonym and antonym blocks.

### MRI acquisition and processing

Participants underwent MRI scanning on a 3 Tesla Siemens Trio Tim System with a 12-channel head coil before and after the intervention; however, only the pre-intervention scans were analyzed in this study given our hypotheses regarding correlations between baseline brain modularity and cognitive gains. The anatomical scan consisted of T1-weighted MPRAGE images acquired with the following parameters: GRAPPA acceleration factor 2, voxel size = 0.9 × 0.9 × 0.9 mm, *TR* = 1,900 ms, *TI* = 900 ms, *TE* = 2.32 ms, flip angle = 9°, FoV = 230 mm. To analyze network properties during a task-free “resting state,” a 6-min functional scan was obtained using a T2^*^-weighted echoplanar imaging (EPI) pulse sequence with the following parameters: GRAPPA acceleration factor 2, 180 volumes, in-plane resolution = 3.4 mm^2^, *TR* = 2,000 ms, *TE* = 25 ms, flip angle = 80°, 35 4 mm ascending slices, no slice gap. Participants were instructed to lie still with their eyes closed.

Brain extraction from anatomical scans was performed with Advanced Normalization Tools (ANTs; Avants et al., 2010, 2011) using the Kirby/MMRR template (Landman et al., 2011). When this skull-stripping procedure failed, brain extraction was instead performed using the IXI template (Heckemann et al., 2003; Ericsson et al., 2008). The skull-stripped anatomical images and raw functional images were preprocessed through the Configurable Pipeline for Connectomes (CPAC; Giavasis et al., 2015). Anatomical images were registered to the MNI152 template (Fonov et al., 2009) using ANTs and segmented into gray matter (probability threshold = 0.7), white matter (probability threshold = 0.98) and cerebrospinal fluid (CSF; probability threshold = 0.98) using FSL/FAST (Zhang et al., 2001). Functional images were slice-time corrected, motion-corrected (Friston et al., 1996) and co-registered to the anatomical images. Nuisance signal removal was performed by regressing out the aforementioned motion parameters, signals from the first five components from white matter and CSF voxels (Compcor; Behzadi et al., 2007; Muschelli et al., 2014), and linear and quadratic trends. Signals were bandpass filtered at 0.009–0.08 Hz. Participants whose resting state scan contained (1) more than 10% of volumes with framewise displacement (FD) greater than 0.5 mm (*N* = 23) or (2) maximum absolute displacement greater than 4.0 mm were excluded from subsequent analyses (additional *N* = 12). One participant was excluded because structural abnormalities caused anatomical-to-MNI registration to fail (spatial warping) during preprocessing, such that we could not reliably extract ROIs.

### Functional connectivity and modularity analyses

Functional scans were warped to the MNI template and parcellated into 264 regions of interest (Power et al., 2011). Due to uneven partial coverage of the cerebellum across subjects in the functional data, we excluded the four cerebellum module ROIs prior to analysis. Eight additional ROIs were excluded due to lack of functional coverage in at least one participant, leaving a total of 252 ROIs. For each individual, time series from all voxels within each ROI were averaged together. Average ROI time series were correlated between each pair of ROIs (Pearson's coefficient), and the resulting ROI-to-ROI correlation matrices were Fisher z-transformed. Matrices were binarized over a range of connection density thresholds (costs): 2–10% of all possible connections, in 2% increments, following (Power et al., 2011; Power and Petersen, 2013). These thresholded matrices were used to create unweighted, undirected whole-brain graphs for each participant, from which network metrics were derived using the BrainX (https://github.com/nipy/brainx) and NetworkX Python package (Hagberg et al., 2008). Network modularity was quantified separately for each connection threshold to examine the consistency of results across thresholds. We use the middle 6% threshold for all our primary analyses, but verified effects at the other thresholds (Supplementary Material).

For our primary analysis, we quantified modularity, a network measure that compares the number of connections within modules to the number of connections across modules (Newman and Girvan, 2004). Modularity is defined as$\overset{m}{\underset{i\;=\;1}{\sum }}\left(e_{ii}-a_{i}^{2}\right)$, where *e*_*ii*_ is the fraction of connections that connect two nodes within module *i*, *a*_*i*_ is the fraction of connections connecting a node in module *i* to any other node, and *m* is the total number of modules in the network (Newman and Girvan, 2004). There are multiple methods for identifying network modules. Here, we used a spectral algorithm (Newman, 2006a) to identify the partition that maximizes modularity for each participant at each threshold.

Further, to confirm that our effects were not driven by a specific partitioning algorithm, we also computed modularity using partitions identified in Power et al. (2011) using the Infomap algorithm (Rosvall and Bergstrom, 2008; Fortunato, 2010). Here, every node was assigned to one of thirteen modules (as identified in Power et al., 2011): default mode (DMN), fronto-parietal (FP), cingulo-opercular (CO), salience (Sal), dorsal attention (DAN), ventral attention (VAN), auditory (Aud), visual (Vis), memory (Mem), sensory/somatomotor hand (SM-hand), sensory/somatomotor mouth (SM-mouth), subcortical (Subcort) and a module containing unassigned nodes. The modularity values derived from the Power partition were highly correlated with the modularity values obtained using the spectral clustering partition (all *r* > 0.761, all *p* > 0.001, two-tailed for all five cost thresholds).

### Potential confounds

Before examining the relationship between brain modularity and intervention-related gains, we examined relationships between potential confounding variables and our measures of interest (i.e., baseline modularity and intervention-related gains), including age, in-scanner motion (i.e., frame-wise displacement or FD; Power et al., 2012; Satterthwaite et al., 2012; Siegel et al., 2016), and baseline cognitive performance. All the analyses include only subjects with usable baseline MRI scans, baseline EF scores, and EF gain scores (*N* = 128). If a significant relationship between potential confounding variables and our dependent measures was found, we then used these variables as covariates in our primary analyses examining correlations between modularity and intervention-related gains. For all analyses, we also controlled for age and in-scanner motion (i.e., FD). For all correlation analyses, we computed bias-corrected and accelerated (BCa) confidence intervals (CI) using 5,000 bootstrapped samples.

There is considerable variability in brain volume in older adults (Salat et al., 2004; Raz et al., 2005; Raz and Rodrigue, 2006). Thus, for participants with structural volume data, we also tested whether the pattern of brain-behavior relationships from the network analyses could have been confounded by gross individual differences in brain structure. We extracted measures of brain volume using Freesurfer v5.3 (Dale et al., 1999); http://surfer.nmr.mgh.harvard.edu), which performs segmentation of cortical and subcortical matter using automated and probabilistic algorithms (Fischl et al., 2002, 2004a,b; Desikan et al., 2006). AZB inspected the segmentation output and performed appropriate corrections. Using the anatomical scans obtained at baseline, we obtained measures of total intracranial volume, white matter, and total gray matter volume, described in more detail on the Freesurfer website (https://surfer.nmr.mgh.harvard.edu/fswiki/MorphometryStats). We included estimated intracranial volume as a covariate in volumetric analyses to control for differences in overall brain volume (Jack et al., 1989; Buckner et al., 2004). Since not all participants had high-quality structural scans for volumetric analysis (*N* = 15), we conducted this analysis as a follow-up to the primary analyses of modularity vs. intervention-related gains.

## Results

### Exercise-related changes in cardiorespiratory fitness (CRF)

**Figure 1:**
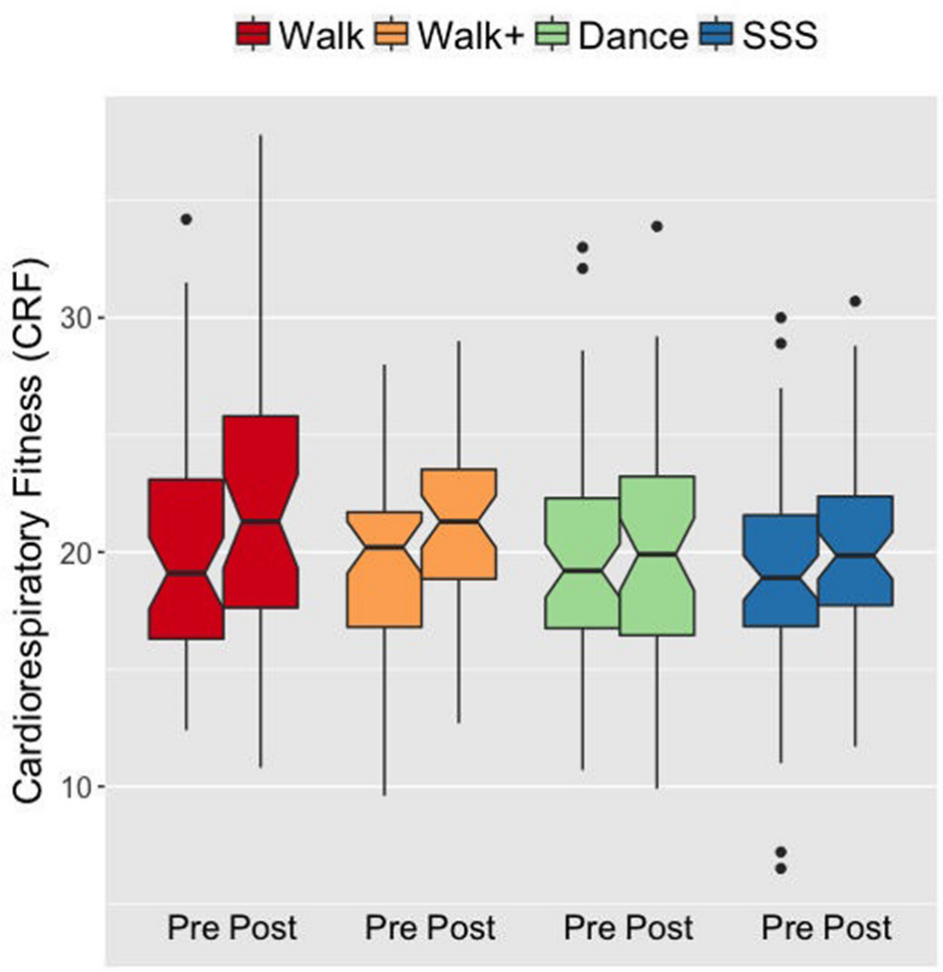
Notched box plots show the distribution of CRF values before and after the intervention. The horizontal line marks the median. The notches extend to ±1.58 IQR/sqrt(n). The upper and lower hinges correspond to the first and third quartiles. The whiskers extend from the hinge to ±1.5^*^IQR of the hinge. IQR, inter-quartile range.

We first verified that the groups demonstrated the expected patterns of fitness improvements. At baseline, the groups did not differ in CRF *F*_(3, 182)_ = 0.199, *p* = 0.897, $\eta_{p}^{2}$ = 0.003. A mixed ANOVA with VO_2_peak scores over time (pre- and post-testing) as a within-subjects factor and group as a between-subjects factor revealed a main effect of time *F*_(1, 182)_ = 21.737, *p* < 0.001, $\eta_{p}^{2}$ = 0.107, and an interaction of group and time *F*_(3, 182)_ = 2.792, *p* = 0.042, $\eta_{p}^{2}$ = 0.044. Follow-up analyses showed that the Walk and Walk+ groups showed greater improvements in CRF compared to the SSS and Dance groups (Figure 1). Separate comparisons of pre- and post-test scores within each group showed significant gains in the Walk and Walk+ groups (both *p* = 0.001, both *d* ≥ 0.539) and marginal gains in the SSS group (*p* = 0.059, *d* = 0.225). There were no significant gains in the Dance group (*p* = 0.345, *d* = 0.062).

### Exercise-related changes in cognitive function

To determine the effects of the exercise intervention on cognitive function and to minimize measurement error and multiple comparison issues in analyzing each test separately, we analyzed cognitive effects at the construct level using composite scores. The creation of composite scores was guided by previous literature (Kane et al., 2005; Salthouse, 2005), correlations (Supplementary Table 1), and a PCA on the baseline test scores (Table 2), which confirmed the grouping of the cognitive tests into categories of vocabulary, episodic memory, perceptual speed, and executive function.

At baseline, the groups did not differ in EF, *F*_(3, 187)_ = 1.191, *p* = 0.315, $\eta_{p}^{2}$ = 0.019, perceptual speed *F*_(3, 185)_ = 0.525, *p* = 0.665, $\eta_{p}^{2}$ = 0.008, episodic memory, *F*_(3, 187)_ = 0.098, *p* = 0.961, $\eta_{p}^{2}$ = 0.002, and vocabulary, *F*_(3, 184)_ = 0.619, *p* = 0.604, $\eta_{p}^{2}$ = 0.010. With the exception of a correlation between vocabulary gain and perceptual speed gain [*r*_(186)_ = 0.152, 95% *CI* [−0.002, 0.304], *p* = 0.039, two-tailed], and between vocabulary gain and EF gain [*r*_(188)_ = 0.147, 95% *CI* [0.011, 0.275], *p* = 0.043, two-tailed], the composite scores of cognitive change scores (i.e., gains) were not significantly correlated with each other (all others *p* > 0.05, two-tailed). Therefore, we conducted separate ANOVAs on the composite gain scores to examine the differential effects of the intervention on cognitive function (Huberty and Morris, 1989).

**Figure 2:**
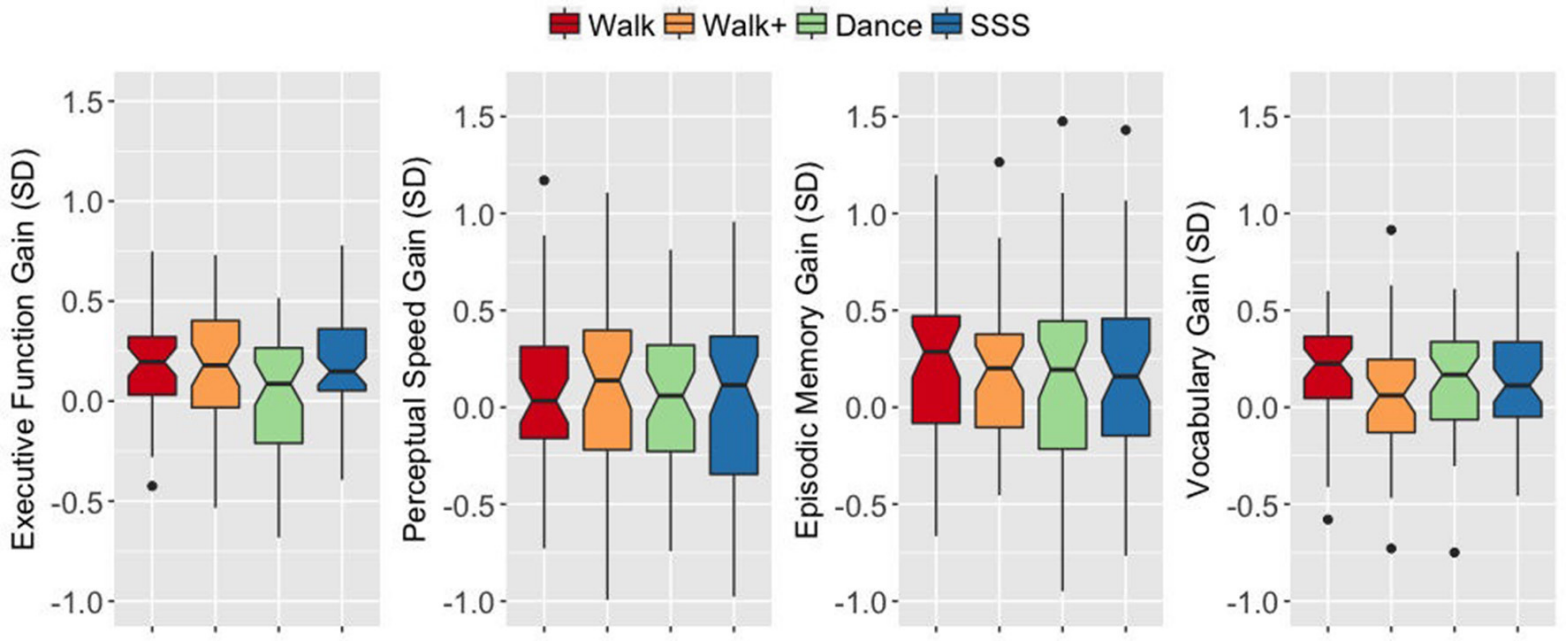
Notched box plots show the distribution of composite gain scores before and after the intervention. The horizontal line marks the median. The notches extend to ±1.58 IQR/sqrt(n). The upper and lower hinges correspond to the first and third quartiles. The whiskers extend from the hinge to ±1.5^*^IQR of the hinge. IQR, inter-quartile range.

An ANOVA on the EF gain score with intervention group as a between-subjects factor yielded a significant group effect *F*_(3, 187)_ = 3.899, *p* = 0.010, $\eta_{p}^{2}$ = 0.059. The Walk, Walk+ and SSS groups showed greater EF gains compared to the Dance group (all *p* < 0.05 for each group vs. Dance, Dance vs. Walk+ is *p* < 0.10 with Bonferroni correction; Figure 2). Tests for significance of gain scores (i.e., test against a comparison value of zero) separately in each group showed significant EF gains in the Walk, Walk+ and SSS groups (all *p* < 0.001), but not in the Dance group (*p* = 0.703). Since baseline EF was moderately correlated with EF gain, *r*_(189)_ = −0.140, 95% *CI* [−0.255, −0.026] *p* = 0.053, two-tailed, we verified that the group effect in EF gain remained significant after controlling for baseline EF, *F*_(3, 186)_ = 3.498, *p* = 0.017, $\eta_{p}^{2}$ = 0.053. No group effects were observed for gains in perceptual speed, *F*_(3, 185)_ = 0.129, *p* = 0.943, $\eta_{p}^{2}$ = 0.002, episodic memory, *F*_(3, 187)_ = 0.086, *p* = 0.968, $\eta_{p}^{2}$ = 0.001, and vocabulary, *F*_(3, 184)_ = 1.376, *p* = 0.251, $\eta_{p}^{2}$ = 0.022 (Figure 2).

### Relationship between fitness and cognitive effects

Given that the groups that improved in EF were also those that showed larger CRF gains (i.e., Walk, Walk+, SSS groups), we tested whether the degree of CRF improvement was related to EF improvement. Across the whole sample with CRF data and behavioral data, there was no significant relationship between CRF gain and EF gain, *r*_(184)_ = 0.104, 95% CI [−0.045, 0.251], *p* = 0.157, two-tailed, even when excluding the Dance group which did not show CRF and EF gains, *r*_(138)_ = 0.080, 95% CI [−0.045, 0.251], *p* = 0.352, two-tailed. The correlations were also not significant within each group (all |*r*| <0.25, all *p* > 0.05, two-tailed).

### Examination of potential confounds

Across the whole sample with quality MRI data, we first examined relationships between group assignment (i.e., to confirm that groups did not differ in baseline characteristics), potential confounding variables (i.e., age, years of education, mean FD) and our measures of interest (i.e., baseline modularity and EF gain). In the case of a non-significant relationship between variables when analyzing the whole MRI sample, we also verified that the relationship was not significant when analyzing each group separately, as the primary analyses of baseline modularity and EF gain were conducted within group.

Age did not differ across groups (Table 1), but was significantly correlated with baseline modularity, *r*_(126)_ = 0.239, 95% CI [0.102, 0.370], *p* = 0.007, two-tailed, and was not correlated with EF gain, *r*_(126)_ = −0.008, 95% CI [−0.211, 0.197], *p* = 0.932, two-tailed. We verified that there was no significant relationship between age and EF gain within each group (all |*r*| <0.314, all *p* > 0.097, two-tailed).

Years of education did not significantly differ across groups (Table 1), even after accounting for age *F*_(3, 123)_ = 2.117, *p* = 0.101, $\eta_{p}^{2}$ = 0.049. Education was not significantly correlated with baseline modularity, *r*_(126)_ = −0.031, 95% CI [−0.183, 0.124], *p* = 0.730, or EF gain, *r*_(126)_ = −0.041, 95% CI [−0.216, 0.137], *p* = 0.649, even after accounting for age, and when examining within each group separately (all |*r*| < 0.295, all *p* > 0.101, two-tailed).

Mean FD did not differ across groups, *F*_(3, 124)_ = 0.938, *p* = 0.425, $\eta_{p}^{2}$ = 0.022, even after controlling for age, *F*_(3, 123)_ = 0.935, *p* = 0.426, $\eta_{p}^{2}$ = 0.022. Mean FD was not correlated with baseline modularity, *r*_(126)_ = −0.087, 95% CI [−0.272, 0.092], *p* = 0.328, two-tailed or with EF gain, *r*_(126)_ = 0.039, 95% CI [−0.138, 0.197], *p* = 0.666, two-tailed, even after controlling for age (all |*r*| <0.122, all *p* > 0.173, two-tailed). When inspecting these relationships within each group however, we found a trending relationship between mean FD and modularity in the Walk group, *r*_(27)_ = −0.359, 95% CI [−0.666, 0.005], *p* = 0.056, two-tailed.

Baseline modularity differed across groups, *F*_(3, 124)_ = 4.628, *p* = 0.004, $\eta_{p}^{2}$ = 0.101, even after accounting for age, *F*_(3, 123)_ = 4.495, *p* = 0.005, $\eta_{p}^{2}$ = 0.099, with the Walk+ group showing significantly lower baseline modularity compared to the SSS (*p* = 0.005) and Dance (*p* = 0.011) groups, but not compared to the Walk group (*p* = 0.116).

Lastly, given previously documented relationships between modularity and cognitive function (Kitzbichler et al., 2011; Stevens et al., 2012; Stanley et al., 2014; Sadaghiani et al., 2015), we examined whether baseline modularity was related to baseline EF. Across the whole MRI sample, there was no significant relationship between baseline modularity and baseline EF, *r*_(126)_ = 0.023, 95% CI [−0.167, 0.207], *p* = 0.798, two-tailed, even after accounting for age and/or mean FD and examining each group separately (all |*r*| < 0.319, all *p* > 0.098, two-tailed). Thus, potential relationships between modularity and EF gains cannot be attributed to correlations between modularity and EF performance at baseline.

The above results were similar when using modularity values derived from other thresholds and when using modularity values derived from the Power partition (see Supplementary Material). Thus, given these findings that age and mean FD showed some relationship with modularity, and given that baseline EF was moderately related to EF gain, we used age, mean FD and baseline EF as covariates in the primary analyses of modularity and exercise-related gains.

### Relationship between baseline modularity and exercise-related gains

**Figure 3:**
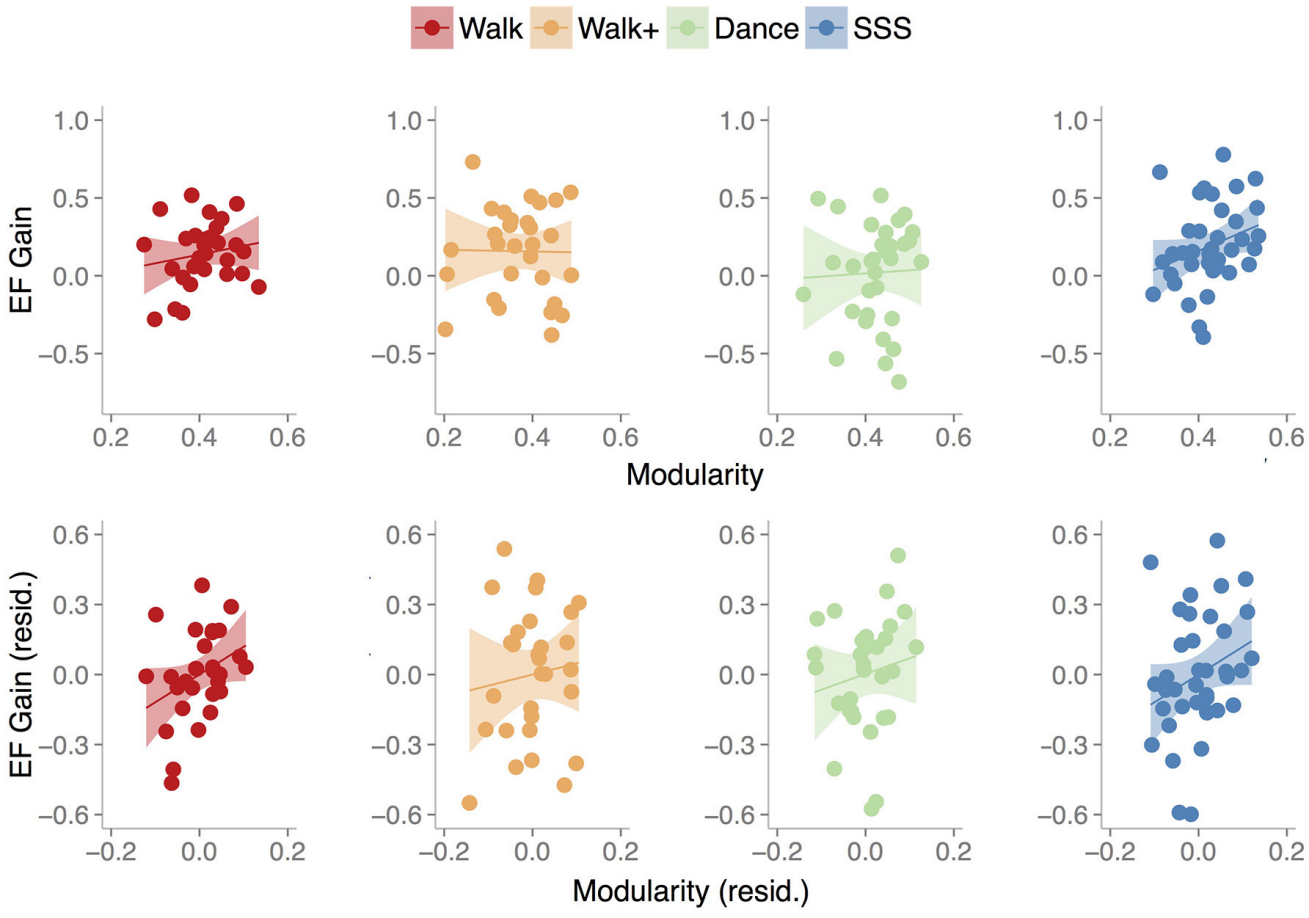
Scatterplots show the relationship between baseline modularity (6% threshold) and executive function gain in each group, without controlling for any other factors **(top)** and after controlling for age, mean FD and baseline EF **(bottom)**. Shaded areas represent 95% confidence region of the regression line.

We next examined the relationship between baseline modularity and intervention-related effects on EF, having confirmed EF and CRF improvements in the Walk, Walk+ and SSS groups (Figure 3). For each group, we first performed linear regression analyses with EF gain as the dependent variable, age, mean FD and baseline EF as covariates, and independent variables of baseline EF, baseline modularity, and an interaction term of baseline EF and baseline modularity. Importantly, the interaction term was included to test whether the relationship between baseline modularity and EF gain was moderated by baseline EF (i.e., whether the modularity-gain relationship was stronger in high or low performing individuals at baseline).

The model (Table 3) with all three terms and covariates was significant in the Walk [*R*^2^ = 0.450, Adjusted *R*^2^ = 0.330, *F*_(5, 23)_ = 3.756, *p* = 0.012] and Walk+ groups [*R*^2^ = 0.375, Adjusted *R*^2^ = 0.239, *F*_(5, 23)_ = 2.762, *p* = 0.043].

**Table 3:** Multiple linear regression models.

	**Walk**		**Walk**+		**Dance**		**SSS**	
	***B* 95% CI**	***p***	***B* 95% CI**	***p***	***B* 95% CI**	***p***	***B* 95% CI**	***p***
Intercept	0.156 [0.090, 0.221]	<0.001^***^	0.173 [0.067, 0.263]	0.001^**^	0.030 [−0.069, 0.143]	0.594	0.190 [0.103, 0.286]	<0.001^***^
Age	−0.021 [−0.032, −0.003]	0.022^*^	−0.041 [−0.073, −0.004]	0.022^*^	−0.009 [−0.051, 0.033]	0.582	0.004 [−0.022, 0.023]	0.710
Mean FD	1.036 [−0.248, 2.226]	0.037^*^	−0.560 [−1.686, 0.488]	0.277	0.778 [−0.403, 2.242]	0.196	0.166 [−1.015, 1.352]	0.747
Baseline EF	−0.011 [−0.124, 0.082]	0.812	−0.190 [−0.434, −0.025]	0.067~	−0.155 [−0.289, 0.101]	0.113	0.007 [−0.113, 0.146]	0.929
Modularity	1.823 [0.343, 3.309]	0.006^**^	0.445 [−1.127, 1.909]	0.553	−0.064 [−2.496, 1.454]	0.951	1.182 [−0.518, 2.624]	0.114
Baseline EF × Modularity	−2.365 [−4.038, 0.197]	0.005^**^	−5.007 [−8.738, −1.301]	0.003^**^	1.406 [−1.730, 5.369]	0.267	−0.261 [−2.612, 1.584]	0.802

*** *p < 0.001*,

** *p < 0.01*,

* *p < 0.05*,

~ *p < 0.10*.

In the Walk group, age, mean FD, modularity, and the interaction term of modularity and baseline EF were significant predictors of EF gain (Table 3). Critically, modularity positively predicted EF gain, while the interaction showed that individuals with lower baseline EF showed a stronger relationship between modularity and EF gain.

In the Walk+ group, age and the interaction term of modularity and baseline EF were significant predictors of EF gain, with baseline EF as a marginal predictor (Table 3). Similar to the Walk group, individuals with lower baseline EF showed a stronger relationship between modularity and EF gain.

In the SSS group, the full model was not significant [Table 3; *R*^2^ = 0.094, Adjusted *R*^2^ = −0.048, *F*_(5, 32)_ = 0.661, *p* = 0.656]. Modularity was not a significant predictor, although it explained the most variance and was related to EF gain in a similar positive direction. Given that there were no significant predictors in the full model, we performed a reduced model with only baseline modularity. This model was marginally significant [*R*^2^ = 0.083, Adjusted *R*^2^ = 0.057, *F*_(1, 36)_ = 3.246, *p* = 0.080], with modularity marginally related to EF gain (*B* = 1.218, *p* = 0.080, BCa 95% CI [−0.439, 2.276]).

As expected, in the Dance group, the full model [Table 3; *R*^2^ = 0.217, Adjusted *R*^2^ = 0.066, *F*_(5, 26)_ = 1.437, *p* = 0.244], and a reduced model with only baseline modularity [*R*^2^ = 0.002, Adjusted *R*^2^ = −0.032, *F*_(1, 30)_ = 0.045, *p* = 0.833] were not significant, with no factor emerging as a significant predictor.

In summary, we find that baseline modularity was related to EF gains in groups that showed training-related gains. For illustrative purposes, Figure 3 shows the relationship between baseline modularity and EF gain with and without controlling for age, mean FD and baseline EF.

### Controlling for individual differences in brain volume

Age-related differences in white and gray matter volume loss may influence brain function (Persson et al., 2006; Chadick et al., 2014; Pudas et al., 2017), functional connectivity patterns (Meunier et al., 2014), and in turn, the pattern of brain-behavioral results we find here. On the sample of participants with high-quality anatomical data, we ran partial correlation analyses of baseline modularity and EF gain within each of the four groups (one-tailed tests to confirm initial results), controlling for estimated intra-cranial volume, gray matter volume, and white matter volume in addition to age, mean FD and baseline EF. Critically, the pattern of relationships remained the same, Walk: *rp*_(16)_ = 0.369, 95% CI [−0.339, 0.884], *p* = 0.066; Walk+: *rp*_(20)_ = 0.098, 95% CI [−0.519, 0.628], *p* = 0.331; SSS: *rp*_(25)_ = 0.408, 95% CI [−0.024, 0.709], *p* = 0.017, Dance: *rp*_(20)_ = −0.017, 95% CI [−0.496, 0.635], *p* = 0.469, suggesting that individual differences in brain volume did not contribute to the relationship between baseline modularity and EF gain.

### Exploratory analyses: sub-network contribution to relationship between baseline modularity and training-related gains

Brain modules show distinct age-related connectivity changes (Chan et al., 2014), and modularity in the association systems (DMN, FP, CO, Sal, DAN, VAN) has been found to drive the correlation between global modularity and training-related gains (Gallen et al., 2016). Given this, we examined whether specific networks contribute to the modularity vs. gain relationship. Similar to previous findings, sensory-motor modularity was higher than association cortex modularity both when analyzing the whole sample, *t*_(127)_ = 24.954, *p* < 0.001, and each group separately (all *p* < 0.001). We then examined the contribution of each sub-network to the modularity vs. EF gain relationship. For these analyses, we performed partial correlation analyses with age, mean FD and baseline EF as covariates. To reduce the number of analyses, we combined the three groups (Walk, Walk+ and SSS) given the similarity in their intervention-related effects.

Across the three groups, EF gain was marginally correlated with baseline association sub-network modularity *r*_(91)_ = 0.159, 95% CI [−0.053, 0.346], *p* = 0.064, one-tailed, but not sensory-motor cortex modularity *r*_(91)_ = 0.003, 95% CI [−0.188, 0.209], *p* = 0.488, one-tailed. Given the trending relationship between EF gain and association modularity and previous findings (Gallen et al., 2016), we examined the relationship between EF gain and each association sub-network. After Bonferroni correction however, none of the six modules showed a significant relationship with EF Gain (Supplementary Material).

We also quantified module segregation (Chan et al., 2014), defined as (Z_w_-Z_b_)/Z_w_, where Z_w_ is the average Fisher-transformed correlation between nodes in the same module (within-module connectivity) and Z_b_ is the average Fisher-transformed correlation between nodes in a module to nodes in any other module (between-module connectivity). Importantly, this metric retains the weights of all connections (lower than 2–10% of connections). Given previous findings, we focused our analyses on the association cortex modules. When controlling for age, mean FD, and baseline EF, whole-brain segregation and association module segregation were not significantly related to EF gain, although the results were in the same direction as the modularity results (Supplementary Material).

## Discussion

We examined whether baseline brain network modularity predicts cognitive improvements in older adults after an exercise intervention. We found that in the groups that showed gains in fitness and cognitive function (Walk, Walk+, and SSS), higher baseline brain modularity predicted greater gains in executive function, even after accounting for individual differences in baseline performance, age, in-scanner motion, and individual differences in brain volume. These results parallel findings in TBI patients (Arnemann et al., 2015), older adults (Gallen et al., 2016), and young adults who underwent cognitive training (Baniqued et al., 2015). Given that we find a similar relationship between modularity and cognitive gains after an exercise intervention in older adults suggests that the predictive power of brain modularity may be generalizable across populations and interventions aimed to enhance executive function. Moreover, these findings point to the potential of global network properties to capture individual differences in neuroplasticity.

### Modularity and exercise-related gains in executive function

Our findings demonstrating a relationship between baseline brain network modularity and EF improvements with exercise training add to a series of studies that find a similar relationship with cognitive gains from cognitive training interventions (Arnemann et al., 2015; Baniqued et al., 2015; Gallen et al., 2016). Importantly, the current study shows that the pattern of results holds after controlling for factors such as baseline cognitive performance, age, and individual differences in brain volume—the latter of which can present a confound, especially when analyzing measures of brain function in older adults, who show considerable variability in age-related atrophy and lesions (Hedden et al., 2012; Grady, 2013). In the current study, the modularity-gain correlations were found in two (Walk, SSS) out of the three groups that showed some improvement in CRF and EF. In the Walk and Walk+ groups, the modularity-gain relationship was moderated by baseline EF, which together with previous findings in older adults (Arnemann et al., 2015; Gallen et al., 2016) underscores the utility of the network modularity measure in lower-performing individuals. These results suggest that the two measures of baseline performance and modularity together may be a better predictor of training-related gains than either alone.

The relevance of the modularity metric in neuroplasticity, specifically, in predicting response to an intervention, can be linked back to computational models showing that modular networks more rapidly reconfigure in response to new environments (Kashtan and Alon, 2005; Clune et al., 2013; Tosh and McNally, 2015), such that reorganization is more efficiently achieved by slight modifications within and between relatively specialized modules than by a large-scale overhaul of a highly interdependent network. Moreover, individuals with disrupted modular brain organization (Fornito et al., 2015), such as those with focal lesions to brain regions important for between-module connectivity (Nomura et al., 2010; Gratton et al., 2012; Warren et al., 2014) show widespread cognitive dysfunction and thus underscore the role of a modular structure in enabling brain processes that support a wide range of behaviors. In a recent study, individuals who scored higher on general intelligence tests tended to show smaller functional connectivity changes between a “resting state” and task performance states (Schultz and Cole, 2016), suggesting that they adapt more efficiently to task demands. In this sense, the architecture of brain networks at rest guides the connectivity patterns that emerge during the performance of various tasks. Indeed, modularity measured during “resting states” has been found to predict working memory performance (Stevens et al., 2012), and stimulus detection in a perceptual task (Sadaghiani et al., 2015). Taken together, these findings suggest that an “optimally” organized network requires less reorganization to be receptive to new input encountered during learning or training, or to capitalize from intervention-related changes in brain function. In the context of the current study, a more modular brain network may potentiate the rehabilitative and protective effects of physical exercise on the aging brain. In fitness interventions, for example, exercise-associated up-regulation in neurotrophic factors has been related to greater exercise-related changes in brain connectivity (Voss et al., 2013a). Given previous findings and the results of the current study, an optimal network for intervention-related cognitive gains is modularly organized at rest, with a balance of within-module connections that support local processing and across-module connections that support global processing (Meunier et al., 2009, 2010). Indeed, recent studies have shown that increased brain modularity post-therapy correlated with greater speech improvement in aphasic patients (Duncan and Small, 2016), and that greater structural modularity prior to carotid artery intervention predicted reduced risk of cognitive decline after carotid intervention (Soman et al., 2016). Additionally, connectivity measures obtained during preclinical stages, when combined with biomarkers such as amyloid-beta, have been shown to predict later cognitive decline (Buckley et al., 2017), suggesting that these metrics have the potential to provide actionable information when clinical symptoms have yet to manifest.

We found that modularity predicted training gains, beyond the baseline behavioral EF measure. This is a promising finding given that behavioral or cognitive measures may be confounded in certain populations (Gabrieli et al., 2015), such as in older adults, where factors such as mobility or visual acuity interact with task performance. While typical behavioral measures may not reliably distinguish between individual differences in cognitive ability, brain network measures provide a way to gauge training responsiveness. Although this study involved a fairly large sample, functional connectivity was assessed during a relatively short resting-state scan. More reliable measures and more information regarding network structure, particularly in higher performing individuals, may be gleaned from a longer scan period (Birn et al., 2013; Laumann et al., 2015; Gordon et al., 2017). Nonetheless, the pattern of higher baseline modularity predicting intervention-related cognitive gains is now consistent across four studies (Arnemann et al., 2015; Baniqued et al., 2015; Gallen et al., 2016). Using brain network measures in combination with behavioral, demographic, lifestyle, and other brain measures could also help customize intervention protocols to maximize effectiveness, especially in the context of dose-response relationships, for example by increasing the intensity, frequency, or duration of exercises, or including pre-intervention lifestyle or behavioral protocols geared to promote or maintain optimal levels of brain modularity. Nonetheless, future work may identify behavioral measures that are sensitive to the information captured by network measures; the relationship between baseline modularity and future behavior (i.e., training gains) suggests that modularity may be reflected in baseline behavior to some extent, a brain characteristic that the current study's behavioral measures are not designed to capture. In addition, more work is needed to examine the mechanisms in which a modular architecture interacts with changes in neural and vascular function to enable benefits from cognitive and fitness interventions, and whether such interventions lead to changes in brain modularity. In the current study, we found a marginal correlation between EF gain and baseline association cortex modularity, which suggests that association sub-networks drive the relationship between baseline modularity and EF gain, similar to our previous study (Gallen et al., 2016). Relatedly, association sub-networks have also been shown to increase in functional connectivity after a physical exercise intervention (Voss et al., 2010b), concomitant with improvements in EF.

In our dataset, we found a positive correlation between age and baseline modularity, unlike previous studies that found lower modularity in older adults compared to young adults (Meunier et al., 2009; Betzel et al., 2014; Song et al., 2014; Geerligs et al., 2015). Importantly, our study only included older adults, whereas reductions in modularity are typically found when comparing older and young populations. In addition, some studies show no correlation between modularity and age within older adults (Geerligs et al., 2015; Gallen et al., 2016), no difference in modularity *per se* when comparing young and old adults (Meunier et al., 2009) and observations that modularity variability was higher in older adults (Song et al., 2014). Moreover, our older adult sample may not be representative of the general population, as participants were relatively healthy and free of major health incidents despite being generally inactive or sedentary prior to participating in the study. Notably however, the relationship between baseline modularity and training gain in the current study remained even after accounting for age.

Neurovascular coupling is an important issue to consider when conducting fMRI studies in older adults, where age-related vascular changes may lead to age-related BOLD differences in the absence of “true” neural differences (D'Esposito et al., 2003; Samanez-Larkin and D'Esposito, 2008). The current study however, does not compare heterogeneous groups (e.g., young vs. old, low-fit vs. high-fit)—all participants were low-fit but relatively healthy older adults, and all analyses controlled for age. Moreover, across the whole sample, baseline VO_2_ and baseline modularity were not significantly correlated (all |*r*| <0.067, all *p* > 0.457, two-tailed), even after controlling for mean FD. In addition, controlling for baseline VO_2_ in the modularity vs. training gain analyses does not change the results. Future studies can include taking into account indicators of cerebrovascular health such as cerebral blood flow (Brown et al., 2010; Zimmerman et al., 2014) to determine whether and/or to what extent it relates to connectivity measures. In the current study, we controlled for measures such as age, medication, and structural brain measures to examine the potential effects of confounds common to studying an older population. Nonetheless, methodological considerations such as the use of population-specific brain templates may help increase the reliability of brain measures (Buckner et al., 2004).

### Fitness and cognitive gains after exercise intervention

The cognitive improvements in the current study are similar to previous studies that find the largest gains in executive function after aerobic exercise training (Colcombe and Kramer, 2003; Guiney and Machado, 2013; Voss et al., 2013c; Kelly et al., 2014). Here, we used a composite score to analyze training effects instead of assessing group by time interactions in each cognitive task, which can be problematic given the multitude of tasks which requires multiple statistical comparisons. Nonetheless, it is possible that the cognitive effects of the current intervention are driven by specific tasks. For example, the task-switching and spatial working memory tasks in the current study are similar to previous tasks that are sensitive to fitness-related improvements (Hawkins et al., 1992; Kramer et al., 2001; Colcombe and Kramer, 2003; Erickson et al., 2011). On the other hand, improvements in reasoning tasks have been less studied in fitness interventions, although aerobic-related gains in visuo-spatial processes have been documented in younger populations (Stroth et al., 2009; Monti et al., 2012), and improvement in reasoning skills have been found after cognitive training interventions in older adults (Ball et al., 2002; Willis et al., 2006; Lustig et al., 2009). Moreover, compared to previous studies (Colcombe and Kramer, 2003; Voss et al., 2010b; Erickson et al., 2011), the current intervention lasted only 6 months, and it is likely that larger cognitive effects would result from a longer intervention (Colcombe and Kramer, 2003; Kelly et al., 2014). In addition, aerobic exercise has been shown to improve hippocampal function in animal and human studies (Berchtold et al., 2010; Voss et al., 2013c), increase hippocampal volume in humans (Erickson et al., 2011) and to relate to hippocampal-dependent functions such as spatial memory (Erickson et al., 2011) and relational memory (Chaddock et al., 2010). Given these previous findings, we would have expected exercise-related effects not only in the spatial working memory task, but also in the episodic memory tasks. The null findings of the current study may in part reflect a lack of sensitivity in these relatively brief memory tasks in measuring intervention-related change, but may also stem from comparable effects across the four groups, with similar improvements from the different interventions.

In the current study, the SSS group performed exercises that involved some form of resistance training, which has also shown to be beneficial for executive functioning in older adults when performed at a higher intensity (Liu-Ambrose et al., 2010, 2012). Although the strength portion of the SSS exercises in the current study is not comparable to the intensive strength training regimens of other studies, the similarity in exercise style may present an issue for analyzing the effects of interventions such as these, since strength training exercise and aerobic-walking exercise may benefit cognitive function in both differential and overlapping ways. Thus, “null effects” in terms of a lack of differential improvement (i.e., group by time interaction) in other cognitive domains may instead partly reflect comparable gains from the different types of interventions (in addition to gains attributable to test-retest effects) and contamination effects from self-initiated exercise (Ehlers et al., 2016). The Dance group, despite the cognitive demands thought to be involved in the learning and execution of dance steps, showed the smallest effects post-intervention; the group as a whole did not improve in CRF and showed the smallest changes in cognitive function. These findings may in part stem from the heterogeneity and lack of intensity in the Dance sessions, which varied in form (i.e., type of dance) across sessions, and may have thus failed to consistently and intensively train specific physical and cognitive skills. Indeed, Dance participants perceived their in-class sessions as less intensive (Ehlers et al., 2016). Nonetheless, the Dance intervention in the current study has been shown to improve white matter microstructure in the fornix, with baseline fornix fractional anisotropy correlating with baseline processing speed (Burzynska et al., 2017). This paper focuses on EF and connectivity in gray matter, and it is likely that different brain measures reflect distinct aspects of cognitive function. Moreover, the sixth month duration before pre- and post-testing may not adequately reflect longer-term neural and behavioral effects of each intervention.

EF improvements were not directly related to CRF improvements. Combining the test scores into a composite score may have diluted any relationship between CRF gain and gains in a specific test, but no robust correlations were found when examining the relationship between CRF gain and gain on each test measure. In addition, it is possible that intervention-related gains in CRF *per se* does not lead to cognitive improvements, and that indirect effects of exercise on stress, sleep and overall health lead to positive cognitive outcomes (King et al., 1997; Etnier et al., 2006; Cotman et al., 2007; Bherer et al., 2013; Awick et al., 2015). Furthermore, CRF as measured using VO_2_peak in the current study, indexes an array of bodily functions (Dustman et al., 1984; Etnier et al., 2006; Jain et al., 2010) and may not adequately capture cerebrovascular changes.

## Ethics statement

This study was carried out in accordance with the recommendations of the University of Illinois Institutional Review Board, with written informed consent from all subjects. All subjects gave written informed consent in accordance with the Declaration of Helsinki. The protocol was approved by the University of Illinois Institutional Review Board.

## Author contributions

Conceptualization and study design: PB, CG, MV, EM, AK, and MD. Data collection: PB, MV, AB, CW, GC, KD, JF, DE, ES, and SA. Data analysis: PB and CG. Writing—original draft: PB and CG. Writing—review and editing: PB, CG, MV, AB, JF, DE, ES, EM, AK, and MD.

### Conflict of interest statement

The authors declare that the research was conducted in the absence of any commercial or financial relationships that could be construed as a potential conflict of interest.
