# Supplementary material for: Brain Network Modularity Predicts Exercise-Related Executive Function Gains in Older Adults
Source: Front Aging Neurosci. 2018 Jan 4;9:426. doi: 10.3389/fnagi.2017.00426 (PMC5758542; doi:10.3389/fnagi.2017.00426)
Supplement: Supplementary file 1 [file Presentation1.PDF]

Supplementary Material

Brain network modularity predicts exercise-related executive function gains in older adults

1 Supplementary Tables

Supplementary Table 1. Correlation matrix of baseline test scores

	Digit Symbol	Pattern Comparison	Letter Comparison	Word Recall	Logical Memory	Paired Associates	Shipley Abstraction	Form Boards	Letter Sets	Matrix Reasoning	Paper Folding	Spatial Relations	Word Vocabulary	Picture Vocabulary	Synonym-Antonym	Spatial Working Memory P	Task Switching Bin Score	Task Switching Local Cost (RT)
Digit Symbol	1.00	0.66	0.66	0.33	0.26	0.18	0.41	0.29	0.44	0.36	0.17	0.29	0.22	0.02	0.16	0.36	-0.41	-0.05
Pattern Comparison	0.66	1.00	0.57	0.21	0.20	0.13	0.25	0.35	0.32	0.20	0.21	0.29	0.10	0.07	0.11	0.34	-0.35	0.07
Letter Comparison	0.66	0.57	1.00	0.24	0.20	0.11	0.34	0.28	0.30	0.29	0.09	0.19	0.23	0.10	0.16	0.36	-0.26	-0.08
Word Recall	0.33	0.21	0.24	1.00	0.58	0.61	0.32	0.17	0.29	0.36	0.21	0.10	0.47	0.28	0.46	0.27	-0.14	0.08
Logical Memory	0.26	0.20	0.20	0.58	1.00	0.54	0.40	0.34	0.38	0.46	0.29	0.27	0.45	0.36	0.51	0.21	-0.26	0.06
Paired Associates	0.18	0.13	0.11	0.61	0.54	1.00	0.34	0.19	0.26	0.30	0.31	0.20	0.32	0.26	0.33	0.08	-0.18	0.11
Shipley Abstraction	0.41	0.25	0.34	0.32	0.40	0.34	1.00	0.42	0.67	0.56	0.43	0.49	0.51	0.42	0.47	0.21	-0.29	-0.08
Form Boards	0.29	0.35	0.28	0.17	0.34	0.19	0.42	1.00	0.40	0.50	0.44	0.59	0.26	0.35	0.31	0.27	-0.20	0.12
Letter Sets	0.44	0.32	0.30	0.29	0.38	0.26	0.67	0.40	1.00	0.58	0.34	0.45	0.46	0.33	0.45	0.27	-0.35	-0.04
Matrix Reasoning	0.36	0.20	0.29	0.36	0.46	0.30	0.56	0.50	0.58	1.00	0.45	0.56	0.36	0.34	0.42	0.25	-0.32	0.03
Paper Folding	0.17	0.21	0.09	0.21	0.29	0.31	0.43	0.44	0.34	0.45	1.00	0.57	0.20	0.34	0.27	0.29	-0.14	0.12
Spatial Relations	0.29	0.29	0.19	0.10	0.27	0.20	0.49	0.59	0.45	0.56	0.57	1.00	0.22	0.35	0.25	0.23	-0.21	0.18
Word Vocabulary	0.22	0.10	0.23	0.47	0.45	0.32	0.51	0.26	0.46	0.36	0.20	0.22	1.00	0.60	0.72	0.10	-0.13	-0.01
Picture Vocabulary	0.02	0.07	0.10	0.28	0.36	0.26	0.42	0.35	0.33	0.34	0.34	0.35	0.60	1.00	0.62	0.14	-0.14	0.05
Synonym-Antonym	0.16	0.11	0.16	0.46	0.51	0.33	0.47	0.31	0.45	0.42	0.27	0.25	0.72	0.62	1.00	0.13	-0.07	0
Spatial Working Memory	0.36	0.34	0.36	0.27	0.21	0.08	0.21	0.27	0.27	0.25	0.29	0.23	0.10	0.14	0.13	1.00	-0.18	0.16
Task Switching Bin Score	-0.41	-0.35	-0.26	-0.14	-0.26	-0.18	-0.29	-0.20	-0.35	-0.32	-0.14	-0.21	-0.13	-0.14	-0.07	-0.18	1.00	0.33
Task Switching Local Cost (RT)	-0.05	0.07	-0.08	0.08	0.06	0.11	-0.08	0.12	-0.04	0.03	0.12	0.18	-0.01	0.05	0.00	0.16	0.33	1.00

2 Supplementary Analyses

2.1 Examination of potential confounds

Across the whole sample with quality MRI data, we first examined relationships between group assignment (i.e., to confirm that groups did not differ in baseline characteristics), potential confounding variables (i.e., age, years of education, mean FD) and our measures of interest (i.e., baseline modularity and EF gain).

**Spectral**	2%	4%	6%	8%	10%
Age vs. baseline modularity	R=.127, p=.153	R=.193, p=.029	R=.239, p=.007	R=.269, p=.002	R=.268, p=.002
Education vs. baseline modularity, c. age	R=.039, p=.661	R=.016, p=.859	R=-.009, p=.922	R=-.022, p=.809	R=-.029, p=.749
Mean FD vs. baseline modularity, c. age	R=-.049, p=.582	R=-.088, p=.325	R=-.122, p=.173	R=-.111, p=.214	R=-.102, p=.252
Baseline modularity group effect, c. age	F=2.906, p=.037	F=4.520, p=.005	F=4.578, p=.004	F=4.492, p=.005	F=4.403, P=.006
Baseline EF vs. baseline modularity c. age, mean FD	R=.027 P=.761	R=.074, p=.410	R=.073, p=.415	R=.082, p=.362	R=.076, p=.400
**Power partition**					
Age vs. baseline modularity	R=.165, p=.063	R=.203, p=.021	R=.236, p=.007	R=.250, p=.004	R=.255, p=.004
Educ vs. baseline modularity, c. age	R=.052, p=.558	R=.061, p=.499	R=.056, p=.531	R=.055, p=.536	R=.055, p=.541
Mean FD vs. baseline modularity, c. age	R=-.020, p=.824	R=-.087, p=.329	R=-.126, p=.160	R=-.154, p=.083	R=-.160, p=.072
Baseline modularity group effect, c. age	F=3.388, p=.020	F=4.356, p=.006	F=4.477, p=.005	F=4.209, p=.007	F=4.355, p=.006
Baseline EF vs. baseline modularity c. age, mean FD	R=.020, p=.825	R=.038, p=.671	R=.039, p=.666	R=.046, p=.613	R=.050, p=.576

*All *p*s are two-tailed.

2.2 Relationship between baseline modularity and exercise-related gains: Regression

Spectral: 4%

	Walk		Walk+		Dance		SSS	
	B	*p*	*B*	*p*	*B*	*p*	*B*	*p*
Intercept	0.162	<.001***	0.163	.004**	0.032	.565	0.191	<.001***
Age	-0.020	.021*	-0.039	.042*	-0.009	.548	0.005	.658
Mean FD	1.067	.026*	-0.529	.342	0.716	.225	0.151	.768
Baseline EF	-0.005	.904	-0.202	.068	-0.151	.119	0.009	.902
Modularity	1.704	.004**	0.418	.583	-0.245	.795	1.130	.120
Baseline EF x Modularity	-2.231	.002**	-4.090	.011*	1.763	.154	-0.103	.916

Spectral: 8%

	Walk		Walk+		Dance		SSS	
	B	*p*	*B*	*p*	*B*	*p*	*B*	*p*
Intercept	0.168	<.001***	0.170	.001**	0.033	.565	0.190	<.001***
Age	-0.020	.034*	-0.045	.016*	-0.008	.638	0.004	.704
Mean FD	0.867	.080	-0.653	.207	0.757	.207	0.148	.778
Baseline EF	-0.024	.632	-0.193	.063	-0.161	.103	0.011	.888
Modularity	1.711	.016*	0.369	.646	-0.411	.726	0.907	.258
Baseline EF x Modularity	-2.371	.008**	-6.025	.002**	1.505	.266	-0.205	.855

Power partition: 4%

	Walk		Walk+		Dance		SSS	
	B	*p*	*B*	*p*	*B*	*p*	*B*	*p*
Intercept	0.159	<.001***	0.152	.010*	0.040	.462	0.191	<.001***
Age	-0.021	.047*	-0.026	.158	-0.007	.650	0.006	.598
Mean FD	0.721	.185	-0.305	.597	0.852	.120	0.173	.749
Baseline EF	0.007	.896	-0.147	.188	-0.122	.196	0.020	.793
Modularity	1.310	.099	0.253	.805	1.045	.373	0.581	.592
Baseline EF x Modularity	-1.382	.102	-3.161	.079	2.748	.114	0.360	.820

Power partition: 8%

	Walk		Walk+		Dance		SSS	
	B	*p*	*B*	*p*	*B*	*p*	*B*	*p*
Intercept	0.165	<.001***	0.150	.009**	0.042	.459	0.193	<.001***
Age	-0.022	.043*	-0.035	.067	-0.008	.605	0.005	.667
Mean FD	0.663	.217	-0.403	.474	0.886	.124	0.257	637
Baseline EF	-0.019	.731	-0.172	.119	-0.132	.180	0.032	.682
Modularity	1.972	.097	1.108	.436	0.819	.613	1.072	.419
Baseline EF x Modularity	-1.986	.113	-5.297	.039*	3.430	.133	0.892	.644

2.3 Relationship between baseline modularity and exercise-related gains: Partial correlations

**Spectral**	2%	4%	6%	8%	10%
Walk	R=.249, p=.110	R=.338, p=.045	R=.342, p=.044	R=.297, p=.070	R=.286, p=.078
Walk+	R=-.074, p=.359	R=.064, p=.377	R=.110, p=.297	R=.080, p=.348	R=.076, p=.356
SSS	R=.244, p=.079	R=.272, p=.057	R=.278, p=.053	R=.203, p=.121	R=.240, p=.083
Dance	R=.157, p=.207	R=.012, p=.475	R=.058, p=.383	R=.006, p=.487	R=-.038, p=.422
**Power partition**					
Walk	R=.183, p=.185	R=.219, p=.142	R=.205, p=.157	R=.214, p=.146	R=.223, p=.137
Walk+	R=-.047, p=.409	R=.036, p=.430	R=.120, p=.279	R=.130, p=.264	R=.168, p=.206
SSS	R=.053, p=.380	R=.097, p=.290	R=.124, p=.238	R=.139, p=.213	R=.164, p=.173
Dance	R=.248, p=.098	R=.225, p=.120	R=.168, p=.192	R=.126, p=.258	R=.136, p=.242

*All *p*s are one-tailed.

2.4 Controlling for individual differences in brain volume

We ran partial correlation analyses of baseline modularity and EF gain within each of the four groups, controlling for estimated intra-cranial volume, gray matter volume, and white matter volume in addition to age, mean FD and baseline EF.

**Spectral**	2%	4%	6%	8%	10%
Walk	R=.306, p=.109	R=.394, p=.053	R=.369, p=.066	R=.285, p=.126	R=.265, p=.144
Walk+	R=-.077, p=.367	R=.054, p=.405	R=.098, p=.331	R=.100, p=.329	R=.103, p=.324
SSS	R=.343, p=.040	R=.383, p=.024	R=.408, p=.017	R=.345, p=.039	R=.385, p=.024
Dance	R=.106, p=.319	R=-.069, p=.380	r=-.017, p=.469	R=-.078, p=.365	R=-.111, p=.311
**Power partition**					
Walk	R=.315, p=.101	R=.299, p=.114	R=.270, p=.139	R=.247, p=.162	R=.261, p=.148
Walk+	R=-.127, p=.287	R=-.049, p=.414	R=.047, p=.417	R=.063, p=.391	R=.118, p=.300
SSS	R=.038, p=.425	R=.125, p=.268	R=.143, p=.238	R=.155, p=.221	R=.184, p=.179
Dance	R=.180, p=.211	R=.149, p=.254	R=.096, p=.335	R=.044, p=.424	R=.047, p=.418

*All *p*s are one-tailed.

2.5 Exploratory analyses: Sub-network contribution to relationship between baseline modularity and training-related gains

Sub-network modularity

In the groups that showed EF gains (Walk, Walk+, SSS), we ran partial correlation analyses of baseline sub-network modularity and EF gain, controlling for age, mean FD and baseline EF.

	2%	4%	6%	8%	10%
Association	R=.135, p=.099	R=.159, p=.064	R=.159, p=.064	R=.161, p=.062	R=.183, p=.039
Sensory-motor	R=-.075, p=.237	R=-.031, p=.385	R=.003, p=.488	R=.022, p=.417	R=.042, p=.344
DMN	R=.173, p=.048	R=.175, p=.047	R=.174, p=.048	R=.173, p=.048	R=.190, p=.034
FP	R=-.026, p=.403	R=-.010, p=.462	R=-0.10, p=.463	R=-0.10, p=.461	R=.013, p=.450
CO	R=.194, p=.031	R=.153, p=.072	R=.122, p=.122	R=.121, p=.125	R=.036, p=.366
Sal	R=-.015, p=.442	R=.038, p=.358	R=.052, p=.311	R=.061, p=.282	R=.108, p=.152
VAN	R=.020, p=.426	R=.023, p=.414	R=-.033, p=.376	R=-.071, p=.250	R=.011, p=.459
DAN	R=-.028, p=.395	R=.061, p=.282	R=.099, p=.172	R=.116, p=.135	R=.087, P=.204

*All *p*s are one-tailed.

Module Segregation

In the groups that showed EF gains (Walk, Walk+, SSS), we ran partial correlation analyses of baseline network segregation and EF gain, controlling for age, mean FD and baseline EF.

	Segregation	Positive Connections Only	Absolute Value of Connections
Whole brain	R=.129, p=.108	R=.100, p=.169	R=.123, p=.119
Association	R=.092, p=.189	R=.118, p=.130	R=.132, P=.104

*All *p*s are one-tailed.
